# Early evolution without a tree of life

**DOI:** 10.1186/1745-6150-6-36

**Published:** 2011-06-30

**Authors:** William F Martin

**Affiliations:** 1 Institut of Botany III, University of Düsseldorf, 40225 Düsseldorf, Germany

## Abstract

Life is a chemical reaction. Three major transitions in early evolution are considered without recourse to a tree of life. The origin of prokaryotes required a steady supply of energy and electrons, probably in the form of molecular hydrogen stemming from serpentinization. Microbial genome evolution is not a treelike process because of lateral gene transfer and the endosymbiotic origins of organelles. The lack of true intermediates in the prokaryote-to-eukaryote transition has a bioenergetic cause.

This article was reviewed by Dan Graur, W. Ford Doolittle, Eugene V. Koonin and Christophe Malaterre.

## Introduction

Biology currently lacks a robust and comprehensive description of early evolution. We should aim to fill that void, but in a language that operates with biology and chemistry, not with branching patterns in phylogenetic trees, versions of which based on informational genes are called the tree of life. Genomes attest unequivocally to the abundance of lateral gene transfer in microbial chromosome history, but current thinking on early evolution is still largely couched in the conceptual framework of trees. When it comes to getting a fuller grasp of microbial evolution, trees might be standing in the way more than they are actually helping us at the moment, because i) the overall relatedness of prokaryotic genomes is not properly described by any single tree, and ii) the relationship of eukaryotes to prokaryotes is also not tree-like in nature because the endosymbiotic origins of organelles introduces lineage mergers and genetic amalgamation into the evolutionary process. If we aim to deliver to science and society a complete picture of early evolution, then at some point we have to incorporate the origin of life into the larger picture of things, too, which means linking microbial evolution to the elements on early Earth. Overall those are fairly tall orders, but we have to start somewhere.

Getting a better picture of early evolution is important for understanding our place in the larger scheme of things. Yet the further back we look in time, the less we know about the course of life's history. The evolutionary history of organisms visible to the naked eye -- plants and animals -- has a recurrently branching phylogeny that can be more or less accurately represented in the mathematical image of a bifurcating tree. Darwin's mechanisms of natural variation and natural selection were inferred from observations of macroscopic life, and those two mechanisms are still sufficient to explain the phylogenetic history of such organisms whose evolutionary process is fundamentally tree-like in nature. Given modern genome sequencing capabilities and computers, the goal of realizing Darwin's vision of a grand natural system *for those groups of multicellular organisms for which he proposed descent with modification*, is merely a matter of time and effort (and we have the fossil record as a helpful system of independent reference to boot). All we have to do is to collect enough data from genomes and refine our tree-building methods sufficiently to bring the structure of the tree of multicellular organisms into focus.

So certain is the conclusion about our ability to decipher the tree of macroscopic life that many an evolutionary biologist excitedly jumps straight to the conclusion that the concept of a grand natural system -- the unity of bifurcating phylogeny and a natural systematics of cladistic variety -- extends to all organisms and all of evolutionary history. As one phylogeneticist once optimistically put it "*All the events of biological evolution are played out somewhere along the branches of phylogenetic trees*" [1]. However, those of us who are trying to understand topics like early evolution, prokaryotic evolution and the prokaryote-to-eukaryote transition, know that evolution among microbial genomes -- our only source of *genetic *evidence (there is chemical and geochemical evidence, too) for early evolution -- has more to it than just sorting out the order of branches. The claim that all of evolution maps to a tree is not true, it only applies to organisms visible to the naked eye, like in Darwin's day. As it concerns our understanding of the evolution of microbial genomes, acceptance of such claims about universal trees upon which all of life's events can be mapped -- or worse, belief in their truth [2] -- is more likely to impair progress in understanding *early *evolution than to promote it (for the evolution of multicellular life, trees are fine).

Life's early history is a process of microbial evolution, where neither the evolutionary events linking the evolution of genes across prokaryotic genomes nor the processes linking prokaryotes to eukaryotes are strictly tree-like in nature [3]. In prokaryote genome evolution, lateral gene transfer (LGT) is an important mechanism of natural variation [4], while the prokaryote-to-eukaryote transition involved the wholesale merger of prokaryotic genomes via endosymbiosis [5-7]. Eukaryotes have meiosis to ensure that genes are only recombined reciprocally among individuals of the same species, prokaryotes lack both such luxurious mating mechanisms and the genetic lineage purity that they confer [8]. Prokaryotes live in a world where plasmids hop around without respect to systematic categories [9], where individual cells are outnumbered 10:1 by alien infectious agents (phage) that can enter their bodies and insert new genes [10]; they live in a world where some individuals, faced with a stressful life situation, cut up their genomes into a thousand little pieces, package the 4.5 kb genome pieces in protein and toss them into the enviroment as gene transfer agents (GTAs) "in hope" that "someone' will be able to use them [11,12]. Fortunately, daily human experience is deviod of analogous processes: we are not constantly surrounded by ten grape-sized gene injectors that are out to convert our internal organs into intruder copies, and when we feel stressed, we do not saw off our toes and offer them to our neighbors.

When we study the long-term evolution of things like birds and bees and flowers and trees, we can confidently lean on the concept of a tree and safely assume that biological events play out along the branches of a bifurcating phylogeny (of populations). But when we study prokaryote evolution, where LGT abounds, or the prokaryote-to-eukaryote transition [13], which centrally involves the origin of mitochondria and gene transfers to the nucleus[12-15], the processes at hand are not tree-like to begin with.

Taught tradition in evolutionary biology implores us to approach, understand and converse on lineage history in terms of trees. But for microbes, trees are not enough if we want to understand genome evolution [16]. Each gene in a given prokaryotic genome has its genealogical tree, yes, but the farther we go back into evolutionary history, the less congruent are the trees of different genes, and the farther we look across prokaryotic diversity, the smaller is the fraction of genes whose distribution across genomes is accounted for via vertical descent. If we want to depict the evolution of prokaryotes, trees are inadequate because they capture only a tiny amount of the underlying evolutionary dynamics. The tree of life as currently defended [17] is more about classification and the search for a tiny minority of genome data that *might be *treelike over some portions of history -- though is it very difficult to show that they *are *treelike [18] -- than it is about understanding microbial evolutionary dynamics. As some of us have said before: microbial evolution and the tree of life are two different things [8].

This realization has (or should have) two consequences. First, it puts an onus upon us to divorce the study of microbial evolution from a *rigid *manifesto of natural systematics, which is simple enough [3,19]. Second, it puts an even heavier onus upon us to react in our methods of investigation of, and our conceptual approach to, microbial evolutionary processes by resorting to the use of mathematical models that are more complex than the simple bifurcating tree. Of course, it is not absolutely imperative that we react. We can, if we so choose, simply pretend that microbial evolution really is tree-like after all [2,17] and impose the bifurcating tree on our thinking at all levels so that we can ask questions of which bifurcations fit our favorite natural categories better, without daring to ask whether the underlying process going on in nature is tree-like to begin with.

If, however, we choose to react with our repertoire of analytical methods, then the obvious solution is to pursue network-based, rather than tree-based, approaches to the study of microbial genome relatedness. In the realm of networks, we can concern ourselves with questions of how to model, in mathematical terms, the microbial evolutionary process -- for example non-directed [20,21] vs. directed [22] networks -- but we do not need to worry immediately about the implications for systematics, which for prokaryotes or phages [23] can hardly be strictly natural anyway, because of lateral transfer. Networks do not confine our thinking to the preconcieved notion that microbial evolution corresponds solely to a process of pure clonal lineage splittings that, over time, generate the patterns of recurrently bifurcating trees.

### The tree of life is only one impediment to a better understanding of early evolution

If we want a full picture of evolutionary history, we have to look all the way back to life's origin. As the major evolutionary transitions, Maynard Smith and Szathmary [24] listed: i) the origins of replicating molecules in compartments (from replicating molecules), ii) chromosomes (from independent replicators), iii) DNA and protein (from RNA), iv) eukaryotes (from prokaryotes), v) sexual populations (from asexual ones), vi) multicellular life (from protists), vii) colonies (from individuals), and viii) human societies (from primate predecessors). Half of the major transitions they identified fall in the realm of early evolution. Lane [25] lists the ten major inventions of evolution as encompassing the origins of i) life, ii) DNA, iii) photosynthesis, iv) eukaryotes, v) sex, vi) motility, vii) sight, viii) warm bloodedness, ix) consciousness and x) death. He also sees half of evolution's greatest inventions within the realm of early evolution. Koonin [26] lists the origins of i) protein folds, ii) viruses, iii) prokaryotic cells, iv) the major prokaryotic groups, v) eukaryotes and vi) animal phyla as the major evolutionary transitions, again mostly falling within the realm of early evolution. That does not necessarily mean that more interesting things happened during early evolution than later, but perhaps that we just wish that we knew as much about early evolution as we know for events later in evolution.

From my perspective, the three most important processes (only two of which are evolutionary transitions) in early evolution are i) the origin of life, ii) prokaryotic evolution, and iii) the prokaryote-to-eukaryote transition. Traditionally, those are the areas where evolutionary biology's greatest weaknesses have been when it comes to providing a fully tangible account of life's history. One might ask: Is it important for evolutionary biology to provide a better understanding of the very earliest history of life? It is arguably one of the most important frontiers facing science, specifically as evolutionary biology interfaces with society. One might also ask: Can we ever understand anything as complex as the origin of life and early evolution? The answer is unquestionably *yes*, the issue is merely *when *we will attain that understanding.

But much like pulling teeth, making progress on these issues can mean that we have to let go of one or the other of our familiar and trusted theories (or elements thereof) from time to time, which can be painful (but like a visit to the dentist, things generally feel better afterwards). The alternative needs to explain more or as much of the same observations while requiring fewer corollary assumptions (we want better teeth, not fewer). That said, three major obstacles to progress in the field of early evolution can currently be identified, views that served well in their day but that now need to go. These impediments concern three traditional and widely held -- but if we think about it in more detail, untenable -- notions about the course of early evolution that are deeply engrained in the minds of scientists and society and that we must abandon altogether if we are to make headway on these central issues. The three conceptual impediments are the notions that: i) life arose from some kind of organic soup, ii) microbial evolution has anything to do with a tree of life or otherwise has the shape of a tree, and iii) the prokayote-to-eukaryote transition occurred before the origin of mitochondria (as opposed to the more easily defendable view of its having ocurred in the wake of the origin of mitochondria).

### Life is a chemical reaction, not stirred organic soup

The very familiar concept that life arose from some kind of organic soup is 80 years old [27-29] and had best be abandoned altogether. The reason is that life is not about the spatial reorganization of preexisting components, it is a continuous chemical reaction, an energy-releasing reaction, and a far-from-equilibrium process. The proposal that life arose through the self-organisation of preformed constituents in a pond or an ice-pore containing some kind of preformed prebiotic broth can be rejected with a simple thought experiment: If we were to take a living organism and homogenize it so as to destroy the cellular structure but leave the molecules intact, then put that perfect organic soup into a container and wait for any amount of time, would any form of life ever arise from it de novo? The answer is no, and the reason is because the carbon, nitrogen, oxygen, and hydrogen in that soup is at equilibrium: it has virtually no redox potential to react further so as to provide electron transfers and chemical energy that are the currency and fabric of life [30-32].

If not soup, what? Life is about redox chemistry, so the site and environment of life's origin should be replete with redox reactions. Alkaline hydrothermal vents provide a good model for understanding early chemical evolution because they have some similarity to living systems themselves. Perhaps similar to some types of hydrothermal vents observable today, such as Lost City [33-36], alkaline vents during the Hadean would have offered a necessary and sufficient redox potential (in the form of the H_2_-CO_2 _redox couple) and catalytic capabilities (in the form of transition metal ions) to permit organic synthesis at a specific location in space and stably over geological time to give rise to the chemical constituents of life and to foster the transition from geochemically contained chemical networks to *bona fide *free-living cells [30-32]. Why, exactly, are alkaline hydrothermal vents conceptually attractive in the origin of life context? There are a number of reasons, many of which are old as the discovery of vents themselves [37,38] but they remain current [39].

One reason concerns compartmentation. Compartmentation from the environment is essential for the transition from inorganic matter to the first living systems [30,31,40,41], and hydrothermal vents are replete with naturally forming microcompartments [30,34], geochemically formed concentrating mechanisms to enable the origin of replicating systems. Let's recall that replication means doubling of mass, hence the precursors for any doubling need to be in steady supply, ideally at their site of synthesis.

A second reason concerns thermodynamics. Life cannot have emerged against the laws of thermodynamics. Excitingly, the overall reaction for the synthesis of the chemical constituents of a microbial cell is exergonic (energy-releasing) at temperatures of around 50-100°C, as recent calculations show [42] for exactly the kind of alkaline hydrothermal vent (chemically similar to Lost City) that some of us have in mind [39]. In other words, the synthesis of life's substance (protein, nucleic acids, cell wall, lipids, etc) from the concentrations of CO_2_, H_2_S, HPO_4_^2-^, NH_4_^+^, H^+ ^and electrons (H_2_) as would be found at the vent ocean interface of such an alkaline hydrothermal system *releases *energy, even without considering main energy metabolic reactions, just considering anabolic cell mass accumulation [42]. That is very important because most people assume that at life's origin one had to add a special kind of energy somehow (for example in the form of lightning), rather than harnessable chemical energy being available naturally, all the time.

In addition there is the nature and availability of that chemical energy to consider. The most obivious source of energy for early chemical and living systems, and the kind considered by Amend and McCollom [42] is molecular hydrogen, H_2_, steming from geochemical processes, just as are observed at Lost City today [36]. Lost City effluent contains about 10 mM H_2 _[36]. H_2_, both from geological sources [43] and from biological sources [44] is very widely used by prokaryotes as a source of electrons and energy today [45]. But H_2 _is so simple that it is often overlooked: a recent review of the possible sources of energy for the origin of life does not mention H_2 _at all [46], even though it is the most abundant source of accesible chemical energy on the early Earth [47] and arguably the far most likely source of energy for the first prokaryotic cells [32,48]. Where does that H_2 _come from? It comes from a process called serpentinization [47,48]. In submarine hydrothermal systems, seawater circulates through the cust. It is drawn into the crust by gravity through cracks in the rock to depths of ~3 km where it is heated to about 150-200°C (because the deep crust is warmer than the surface). The water undergoes a redox reaction with the vast amounts of Fe^2+ ^in the crust. Water is reduced by Fe^2+ ^to H_2_, which ultimately emerges in the hydrothermal effluent, yieding oxidized iron, Fe^3+^, in the form of Fe_3_O_4 _[47,48].

**Figure 1:**
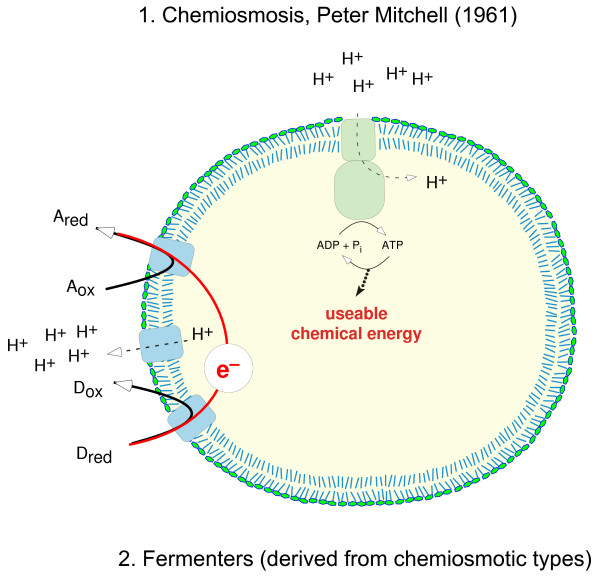
**The two ways that cells conserve energy in the form of ATP (note that some energy conservation in anaerobes involves ferredoxin instead of ATP **[51-53])**: 1) chemiosmosis and 2) substrate-level phosphorylation**. The figure shows a schematic representation of chemiosmotic energy harnessing [50]. A redox reaction (left) is used to channel electrons though plasma membrane-associated proteins and cofactors in such a way that protons are depleted in the cytosol, creating a chemical and electrochemical gradient. Return of the protons to the cytosol occurs through an ATP synthase (ATPase), where ATP is synthesized from ADP and P_i_. D donor, A acceptor, _red _reduced, _ox _oxidized. In rhodopsin-based photosynthesis, the protons are pumped without redox reactions, but in the absence of redox chemistry, no cell can survive. See references [49-53].

Enter the ominous issue of the origin of chemiosmotic energy coupling. The process of serpentinization also makes the effluent alkaline, and Lost City effluent is about pH 10 [33,43]. Why is that significant? Figure 1 depicts the problem. If we look around in biology very hard, and consult very knowledgeable bioenergeticists [49] to make sure, we find that there are only two ways that energy is conserved *in the form of ATP *among life forms that we know: chemiosmosis [50] entailing rotor-stator type ATPases and substrate level phosphorylations. That is very significant. It is also a very carefully worded sentence because a new mechanism of energy conservation has recently been discovered in strict anaerobes that does not involve the phosphoanhydride bond in ATP. It involves the synthesis of reduced ferredoxin -- an energy rich compound -- coupled to a second exergonic redox reaction. The coupling mechanism is called electron bifurcation, because electron pairs are spilt in the reaction mechanism, which entails a flavin cofactor [51-53].

**Figure 2:**
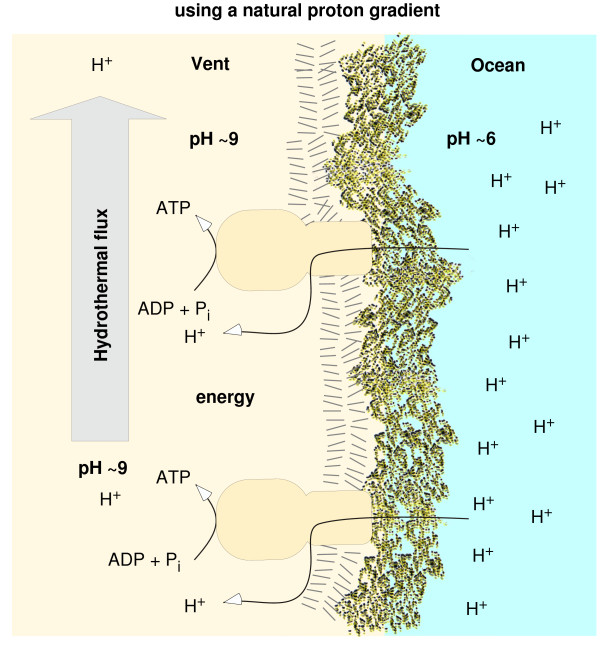
**An alakaline hydrothermal vent harbours a natural proton gradient**. The flux of hydrothermal effluent maintains an alkaline interior. In the presence of appropriate proteins, this source of energy could, in principle, be tapped. The harnessing of naturally preexisting chemiosmotic gradients before the advent of genetically specified mechanisms to generate such gradients would directly explain why ATP synthases of the F-type (eubacteria) and A-type (archaebacteria) are universal and conserved [63], but the mechanisms to generate proton gradients are not. See references [30-32,48].

The only organisms that do not use ATPases for their energy conservation are pure fermenters, who gain their energy through the disproportionation of reduced carbon compounds. But those reduced carbon compounds are made by autotrophs, either photoautotrohs or chemoautotrohs, which always -- without known exception -- use chemiosmotic coupling mechanisms. Moreover, pure fermenters are always derived from chemiosmotic ancestors. Chemiosmosis is the ancestral state of ATP-dependent energy harnessing among free-living cells [54]. Chemiosmotic energy coupling has two components. The first is a membrane-bound multisubunit rotor-stator type ATPase (called F-type in eubacteria and A-type in archaebacteria, the F- and A- types are structurally similar and related). The second is a membrane bound protein-cofactor system that performs a redox reaction of the type D_red _+ A_ox _→ D_ox _+ A_red _(whereby D and A stand for electron donor and acceptor, respectively [50]) the vectorial orientation of whose components across the membrane results in cations, usually protons, being removed from the cytosol and deposited outside the cell, making the inside of the cell alkaline relative to the environment. The origin of this ancestral state of energy harnessing, as universal as the genetic code, is usually but not always [30] disregarded altogether in the early evolution literature, as Mike Russell has repeatedly pointed out. The ion-pumping machinery is extremely variable across prokaryotes, whereas the ATPase is conserved. Which came first? Today, the two components are dependent upon each other to provide a functional unit. In the early evolution of life at an *alkaline *hydrothermal vent, the ATPase (a protein) could have harnessed the naturally preexisting proton gradient (roughly pH 9 inside, roughly pH 6 outside [30,32,48]), at the vent ocean interface (Figure 2). That would directly account for the circumstance that the ATPase and the principle of harnessing ion gradients across the prokaryotic plasma membrane is universal and far more conserved than i) the components that generate the ion gradient and ii) even the chemical constituents of membranes themselves, if we consider the breadth of prokaryote diversity. One might ask how then early chemical systems conserved any chemical energy at all prior to the advent of chemiosmotic coupling (which requires genes and proteins). The suggestion is that H_2_-, CO_2_-, and methyl-dependent (perhaps methylsulfide) substrate level phosphorylations were the initial source of chemical energy, leading to acyl phosphates such as acetyl phosphate that were the first broadly available energy currency for biochemical evolution at the vent up to the level of genes and proteins [32]. Acyl phosphates still have a very broadly distributed and central role in prokaryotic energy metablic processes today [55].

The abiotic (geochemical) synthesis of simple organic compounds at Lost City today provides exciting clues as to what kinds of chemistry the early Earth might have had in store. Lost City effluent contains about 1 mM methane with an isotope signature that indicates an abiotic origin, probably through reduction of dissolved CO_2 _via serpentinizarion [36]. Although a minor component of the methane might be of biological origin, the vast majority is of geochemical origin [56]. Short alkanes, up to pentane, of abiotic origin are also detected, as is (significantly) formate (about 0.1 mM) [57]. This indicates that geochemical processes can indeed produce compounds relevant for biochemistry, very much in line with predictions of the view that life arose in such environments. The smaller amounts of acetate in Lost City effluent (about 0.01 mM) appear at present to be of biological origin [57]. One might like to see evidence for the abiotic synthesis of more complex organic compounds in Lost City efluent, but one needs to recall that the subsurface biosphere is a vast [58] and hungry community, and if anything organic of much direct microbial use is synthesized geochemically, the chances are good that it will be consumed before the effluent reaches the surface. That circumstance (the prokaryotic colonization of the deep subsurface) is perhaps the biggest difference (apart from abundant O_2_, sparse CO_2 _and lacking Fe^2+ ^in modern ocean water) between chemistry at a Lost City type vent today and what might have been going on in a similar system during the Hadean.

The kinds of microbes inhabiting Lost City are mainly methanogens (and anaerobic methane oxidizing forms thereof) [43]; one can only speculate about the possibility of more deeply inhabiting acetogens, based on effluent acetate concentrations [57], and recalling that Lost City effluent is rich in H_2 _but devoid of detectable CO_2 _[43,57]. Methanogens and acetogens generate their ATP chemiosmotically during the process of synthesizing reduced organic compounds for biosynthesis [59,60]. Chemically they have more in common than their genes might suggest [32]. Significantly in my view, both acetogens and methanogens are known that lack cytochromes, and these forms generate their chemiosmotic potential without the help of either quinones or methanophenazine (the functional analogue of quinones in methanogens) [59,61]. Chemiosmotic pumping without the use of quinones or their analogue is generally rare in biology, simpler (requiring many fewer genes) than cytochrome-dependent chains, and arguably the most primitive state of proton-pumping machineries. That the corresponding ion-pumping complexes of cytochrome-lacking methanogens (a methyltransferase, the MtrA-H complex [53,59]) and of cytochrome-lacking acetogens (a ferredoxin:NADH oxidoreductase, the Rnf complex) [61,62] are unrelated is not surprising, as these represent, in this view, independent molecular solutions to the problem of how to generate a proton gradient (how to replace the naturally preexisting one at an alkaline vent) with a chemistry that is specified by genes.

It is also noteworthy that the methanogens (archaebacteria) and acetogens (eubacteria) share chemically related but genetically distinct versions of the acetyl-CoA pathway of CO_2 _fixation and the distinction of living from some of the smallest redox potentials known to fuel free-living cells [63]. The biochemistry of acetogens and methanogens appears to have enough in common with geochemistry at alkaline vents to make the case that they are homologous (related via common ancestry) [32]. Are molecular data in any way compatible with that view? Evidence, albeit based in trees, was presented by Kelley *et al. *[64] "*for a methanogenic origin of the Archaea*". For acetogens (taxonomically, clostridias and relatives, Firmicutes, or low GC Gram positive bacteria) similiar tree-based evidence for their basal position among eubacteria has been presented: "*In our tree, Firmicutes are placed at the earliest division of Eubacteria with 66% BP support, and 33% of remaining BP show at least a subclade of Firmicutes at the earliest division*" [2]. There is no reason to get too excited about that, because the results are based in trees, but it is worth noting that the results come out this way. Genomes from *Clostridia *(and *Thermotoga*) lineages also draw attention to the origin of their ancient traits [65], though not in the context discused here.

The view that the acetyl-CoA pathway is the ancestral state of microbial carbon and energy metabolism suggested an important role for chemically accessible methyl groups, for example methyl sulfide, at the orgin of biochemistry [32]. It has since become apparent that methyl sulfide is an important intermediate in modern microbial interactions involving methanogens [66]. The harnessing of a prexisting proton gradient at the vent ocean interface clearly suggests that protons were the ancestral substrates of ATPases. Mulkidjanian and colleages [67] examined ATPase sequences and came to the conclusion that sodium might be the ancestral substrate, but they also noted that their conclusion is contingent on the premise that the same set of ligands conferring Na^+ ^specifiity over H^+ ^specificity did not arise through convergent evolution. It turns out that "the same set of ligands" boils down to two amino acid substitutions in the membrane portion of the enzyme [67], whereby the convergent emergence of two amino acid substitutions is a commonplace observation even during human mitochondrial DNA evolution [68], so the view that protons were the ancestral substrate of ATPases is compatible with available data. Like the acetyl-CoA pathway, the newly discovered mechanism of energy conservation entailing electron bifurcation and cytoplasmic ferredoxin pools, which is arguably ancient [51], is found in both clostridias [52] and methanogens [53], compatible with the premisses set forth above.

### At depth, the tree of life is not a tree

The second concept about early evolution that we need to abandon is the notion that the overall course of prokaryotic evolution can be accurately described using the mathematical model of a bifurcating tree as a model for the evolution of chromosomes. This point, namely that prokaryotic evolution is not a tree, has been argued often enough in these pages [8] and elsewhere [69]. The nature of the main arguments has not changed much in the meantime. Prokaryote evolution is not treelike because lateral gene transfer (LGT) is a real and prevalent mechanism of natural variation among prokaryotes [3]. Genomes sequences have revealed that over evolutionary time, prokaryotic genomes undergo LGT, the known mechanisms of which entail acquisition through conjugation, transduction, transformation, and gene transfer agents in addition to gene loss [70-72]. This leads to different histories for individual genes within a given prokaryotic genome and networks of gene sharing across chromosomes among both closely and distantly related lineages. In genome comparisons, LGT is traditionally characterized in terms of conflicting gene trees [73,74] or aberrant patterns of nucleotide composition [75]. But in the larger picture of genome evolution, a tree can account for only about 1% of prokaryotic evolutionary history at best [18,76].

If not a tree, what then? Networks should, in principle, be able to more fully uncover the dynamics of prokaryotic chromosome evolution. Networks are currently used to model various aspects of biological systems such as gene regulation [77], metabolic pathways [78], protein interactions [79], conflicting phylogenetic signals [80,81], and ecological interactions [82]. Although it is obvious that a network analysis of gene distributions across prokaryotic genomes should provide a more realistic view of microbial evolution, one that incorporates the contribution of LGT into the process, if we look to the literature, the field is mostly still using trees [83], although evolutionary networks are actively being used as well [9,20-23,84,85], opening up many new and fruitful interdisciplinary avenues of pursuit. The use of networks instead of, and in addition to, trees should lead to a change in the way that microbiologists approach the process of prokaryote genome evolution.

Biologists and philosophers recently inspected the tree of life concept, unearthing a multitude of insights and a plurality of views [86-97]. In that discourse, nobody came out in favor of the concept of a single unified bifurcating tree (and classification system) as the Rosetta stone to life's history, with most contributors pointing to the circumstance that the processes operating in early evolution are not strictly treelike, because of endosymbiosis and because of LGT, which some of us have been saying all along [98,99]. So who, if anyone, is actually defending the tree anymore? The most stalwart defence of the tree of life in recent years came not from biologists, but from a historian [100], and it could be that the most tree-prone among microbiologists would defend the method of using trees to study microbial evolution more vociferously than they might defend any particular tree itself [17]. As leaves and branches in the tree of life come and go, new approaches to the analysis of genome data will continue to emerge, some of which will be based neither on trees nor on networks [101]. It is unlikely that we will still be reading the history of microbial evolution in 20 years time from the order of branching patterns on a tree, as some would like to do it today [2]. Over time, networks will probably come to play an increasingly important role in the study of prokaryote evolution and the prokaryote to eukaryote transition.

### The place of eukaryotes in the bigger picture

Related both to the origin of life issue and the tree of life issue is the place of eukaryotes in the bigger picture of evolution. From the standpoint of the genome sequence comparisons, eukaryotes are genetic chimaeras with archaebacterial type ribosomes in the cytosol, a largely archaebacterial-type information processing machinery and eubacterial-type energy production and metabolic machinery. Lake [102] termed this general functional dichotomy, with its characteristic pattern of conserved sequence similarity, informational and operational genes. This genetic and functional chimaerism in eukaryotes is well documented [5-7,103]. Although there are many theories for the origin of eukaryotes, each designed to account for different aspects of eukaryote biology [reviewed in [104]], only three basic theory varieties address this general pattern of sequence similarity in eukaryotic genomes.

The first is an element of the neomuran theory of eukaryote origins [105,106] called "quantum evolution" [107], a kind of gene-class-and-lineage-specific evolutionary rate fluctuation that is assumed without logical penalties to explain any pattern of observed sequence similarity (or lack thereof). In that view, eukaryotes and archaebacteria arose from actinobacteria less than a billion years ago. Diverging from their common ancestor, the ancestral stem eukaryote became phagocytotic, while the ancestral stem archaebacterium invented things like methanogenesis and everything else that distinguishes archaebacteria from eubacteria (this imaginatively late origin of methanogenesis conflicts with copious geochemical data [32]). The genes showing strong similarity between eukaryotes and archaebacteria -- but not to eubacteria (informational genes) -- underwent a radical kind of fast and furious molecular evolution over a short period of time that created that pattern, while the genes showing strong similarity between eukaryotes and eubacteria -- but not to archaebacteria -- underwent a similarly radical kind of fast and furious evolution in the common ancestor of all archaebacteria [107]. Because the theory operates with rate fluctuations to explain molecular sequence similarites, it can hardly be tested or supported with molecular data. One might complain that genome data link neither eukaryotes nor archaebacteria to actinobacteria, even though that evolutionary transition is only supposed to have occurred 850 MY ago under the theory. However, the nature of the supposed underlying evolutionary process (quantum evolution) erases that sequence similarity trace, for which reason scientific criticism of the theory can be frustrating. As in earlier versions of the theory that derive eukaryotes from cyanobacteria [108], the origin of phagocytosis is seen as the crucial evolutionary invention en route to the eukaryotic state [106], an old idea [109] that still has distinguished supporters today [110]. The earliest-branching eukaryotes in the phagotrophic theory were once called archezoa [111], a taxonomic group that has long since disappeared from the literature, unlike the phagotrophic theory [102] that generated it. While many of the eukaryotic taxonomic groups put forth by Cavalier-Smith have proven robust, his accounts of the prokaryote to eukaryote transition have remained problematic.

In the age of genomes, even supporters of the phagotrophic theory had to account for the circumstance that eukaryotes had archaebacterial informational genes, but preferably without extensive and specific rate fluctuations of gene and protein sequence evolution of the quantum evolution type hard-wired into the theory. Hence a second and popular solution to the origin of informational and operational genes was to suppose that there was some kind of archaebacterial endosymbiont in eukaryote evolution, presumably one that gave rise to the nucleus, usually presumed to have arisen in a eubacterial host. Several variants on this theme emerged [112-118], and continue to emerge [119]. Those theories have at least three major problems in common. First, and in contrast to chloroplasts and mitochondria, the nuclear compartment is in no way homologous to a prokaryotic cell, hence the whole (100 year old) notion that the nucleus was ever an endosymbiont is tenuous to begin with [120]. As with the flagellum [121] or microbodies [122], there is no evidence or homology at all to suggest that the nucleus is descended from an symbiotic bacterium. Second, they derive a cell that has eubacterial genome and eubacterial ribosomes in the cytosol, with the informational genes locked up within the archaebacterial endosymbiont. No formulations of nucleosymbiotic origins to date, even the most recent [119], provides any sound rationale as to how we arrive at a state where the ancestral eubacterial ribosomes and genome in the cytosol are gone and the archaebacterial ribosomes are operating in the cytosol while the archaebacterial chromosomes are retained in the nucleus. Third, theories for an endosymbiotic origin of the nucleus suffer, like the phagotrophic theory, from the circumstance that they all educe, as their crucial intermediate, a eukaryote that primitively lacks mitochondria (an archezoon), thereby predicting that primitively mitochondrion-lacking groups should still be around today. Contrary to that prediction, it has turned out that all eukaryotic groups either once possessed or still possess mitochondria, either in the form of O_2_-respiring mitochondria, anaerobic mitochondria, hydrogen-producing mitochondria (hydrogenosomes) or highly reduced mitochondria (mitosomes) [123-129]. Nucleosymbiotic origin theories keep cropping up in new garb, probably because none of them really account for the observations and remain unconvincing, sometimes even to their proponents [119].

As the third kind of theory that can account for the informational operational classes of eukaryotic genes, some of us have suggested that the host for the origin of mitochondria was simply an archaebacterium. A couple such theories have been proposed [130,131], one of which also directly accounted for the common ancestry of mitochondria and hydrogenosomes and explicitly predicted that *all *eukaryotes lacking typical mitochondria had secondarily lost them [132]. Under that view, called the hydrogen hypothesis, the host was a H_2_-dependent archaebacterium and the endosymbiont was a facultatively anaerobic eubacterium, the common ancestor of mitochondria and hydrogenosomes, whose ability to perform anaerobic H_2_-producing fermentations (in addition to respiration) provided the selective pressure initially associating the symbiont to its host. The archaebacterial nature of the eukaryotic genetic apparatus is readily explained via direct descent from the archaebacterial host, while the eubacterial nature of eukaryotic energy metabolism is directly accounted for via endosymbiotic gene transfers [133] from the genome of mitochondrial endosymbiont to the chromosomes of the host, resulting in the genetic displacement (and chimaeric nature) of many preexisting host pathways [132].

**Figure 3:**
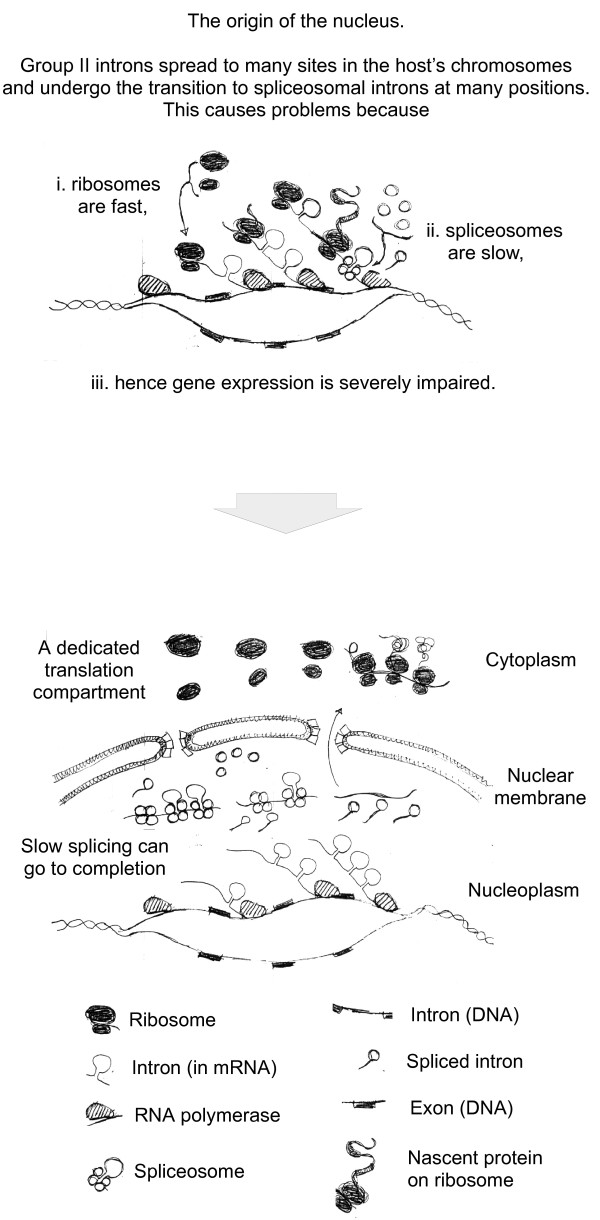
**The origin of spliceosomal introns precipitated the origin of the nuclear membrane in a mitochondrion bearing cell**. See reference [134].

The origin of the nucleus in this mitochondrion-bearing host appears to be linked to the origin of introns and spliceosomes [134] from group II introns of mitochondrial origin that hitchhiked into the host chromosomes during the course or gene transfer. These mobile prokaryotic introns spread to many positions in the genome, and underwent the transition to spliceosomal introns at those ancient eukaryote intron positions. Because ribosomal translation is faster than spliceosomal splicing, the host would only be able to express its (many) intron-containing genes by doing away with the prokaryotic paradigm of cotranscriptional translation (Figure 3). That is, a separation of splicing from translation became imperative for survival (extinction also being an option). Accordingly only such descendants survived that were able to separate splicing from translation by exclusion of ribosomes from the space surrounding active chromatin. Separation in cells usually involves membranes, and this offers a mechanistic rationale that can readily account for the observations that the only cells that ever evolved spliceosomes (eukaryotes) also evolved a nuclear membrane (composed of eubacterial lipids) and had (group II intron containing) mitochondria as well [134]. Compatible with that view, group II introns are indeed still mobile in prokaryotes today [135] and nuclear expression of group II introns yields a splicing behaviour that is very consistent with the predictions of the model [136]. In addition, sequenced eukaryotic genomes are replete with evidence for ongoing gene transfers from organelles to the nucleus today [137,138]. This view for the autogenous origin of the nucleus in a mitochondrion-bearing cell also directly accounts for the observation that eukaryotes have their chromosomes and splicing in the nucleus, their archaebacterial ribosomes in the cytosol and their eubacterial ribosomes in the mitochondrion.

While it is easy to conjure up scenarios for the origin of eukaryotes that are more complicated and involve more partners [112-119] than models involving an archaebacterial host, there are no simpler scenarios, and this simplicity is a virtue of the theory. The only way to invoke fewer cellular partners than an archaebacterial host and a mitochondrial endosymbiont at eukaryote origins [5,129,132] requires reverting to the view that mitochondria did not arise via endosymbiosis [108]. Critics sometimes complain that the eubacterial genes in eukaryotes do not all trace to α-proteobacterial homologues [114,119], but if we incoportare LGT into the bigger picture of genome evolution then it becomes untenable to assume that the ancestor of mitochondria either i) had a pristine genome devoid of "non-α-proteobacterial" genes at the time of endosymbiosis (whatever an α-proteobacteral gene is) or ii) that its free-living descendants never underwent gene donations to, or gene acquisitions from, other prokaryote lineages. In this way, LGT among free-living prokaryotes readily accounts for the apparent eubacterial mosaicism of eukaryotic nuclear genomes via the sample of genes that eukaryotes acquired at the origin of mitochondria [12,99].

Despite the circumstance that prokaryotes can harbour other prokaryotes as endosymbionts [25,139] and despite evidence for multiple origins of phagocytosis in eukaryote evolution [140] some maintain the position that phagotrophy, rather than the origin of mitochondria, just absolutely has to be the first step in the prokaryote to eukaryote transition [106,110]. Whole theories rest on the argument that the only way that a eubacterium can enter another cell to become an endosymbiont is if the host is phagotrophic, sporting staunch quotes: "*Phagocytosis by a protoeukaryote host is the only viable mechanism currently available to explain the origin of the mitochondrion" *[141]. If the phagotrophic argument held any strength, then the existence of bacterial endosymbionts -- in prokaryotes, above, and -- in fungi would be an impossiblity, because fungi do not phagocytose. Well, it turns out that endosymbionts in fungi are common [142] and that in fact, "*Bacteria that inhabit fungi intracellularly, or endosymbiotically, have been described for more than three decades. Endosymbiotic bacterial fungal interactions are ubiquitous and have been documented from all parts of the world*" [143]. So much for (the origin of) phagocytosis being required for the establishment of intracellular endosymbionts.

The extreme stance that eukaryotic cell organisation (and phagocytosis) is ancestral to that in prokaryotes in general [144], is an issue that would appear, in a tree of life mindset, to hinge on branching orders deep in the tree of life [145]. But in this paper we are addressing early evolution without a tree of life and hence wish to approach the issue in a manner that does not hinge upon branches, the abundance of endosymbionts in non-phagocytosing hosts notwithstanding.

For phagocytosis, a cell requires complex machinery of membrane traffic mechanisms: an endoplasmic reticulum, Golgi, actin/tubulin cytoskeleton, cytokinesis, vesicle fusion mechanisms, phagosome formation, exocytosis, etc. That is a considerable level of evolutionary invention, entailing the origin of ~2000 new protein-coding genes [146]. For bioenergetic reasons, the origin of that machinery and its expression as protein requires mitochondria [147]. Prokaryotes have remained prokaryotes for > 3 billion years because they lack mitochondria. The increased energy per host gene that mitochondria confer upon their host by virture of vast bioenergetic membrane surface area, and bioenergetically specialized genomes (mtDNA) to service it [13,147,148], was strictly required to finance, in energetic terms, the origin of eukaryotic novelties. The lack of true intermediates in the prokaryote to eukaryote transition has long been a puzzle, but now it is clear that it has a bioenergetic cause, because prokaryote genome complexity is constrained by membrane bioenergetics [147]. Mitochondria released that constraint and allowed a growth in genome size and complexity and in the number of proteins that a cell can express by four to five orders of magnitude; energy per gene is the key variable [147,148]. Prokaryotes can readily surpass many eukaryotes in terms of cell size [149], but they cannot hold a candle to eukaryotes in terms of true cell complexity. The reason is bioenergetic and boils down to mitochondria, which are the prerequisite to the origin of cell complexity -- hence phagocytosis -- in eukaryotes [147], not *vice versa*. On a good day, that would to put an end to a time-consuming debate regardig the nature of the host that acquired the mitochondrion: prokaryote or phagotroph, the recent history of which was readably summarized by O'Malley [150].

And what about oxygen in eukaryote evolution? Mitochondria afford eukaryotes 10,000 to 100,000 more energy per gene [147], while oxygen affords only a factor of 10-20 [147]. Nonetheless people still seem to think that the advent of oxygen was a decisive event in eukaryote evolution. We all know that O_2 _levels limited animal (but not plant) body size during evolution [151,152]. Under the view of oxygen in Earth history that was current in the 1980s, the rise of atmospheric O_2 _some 2.3 Ga ago was thought to coincide with, and to have provided causal impetus to, the origin of eukaryotes and mitochondria [153]. But since the mid-1990's, a fundamentally different view of Proterozoic ocean chemistry has emerged from isotope geochemistry. In the new and current model of Proterozoic ocean chemistry, the O_2 _that started accumulating in the atmosphere ~2.3 billion years ago began oxidizing continental sulfide deposits via weathering [154], carrying very substantial amounts of sulfate into the oceans, and providing the substrate required for sulfate reducing prokaryotes, hence fueling marine biological sulfate reduction (BSR). Marine BSR became a globally significant process, as evidenced by the sedimentary sulfur isotope record [155]. Marine BSR produces marine sulfide -- H_2_S -- and lots of it. The presence of that sulfide means that the oceans were not only sulfidic during that time, they were also anoxic, both for chemical reasons and because sulfate reducers are strict anaerobes. Although the photic zone (the upper 200 meters or so) was producing oxygen during that time, in the lower photic zone and below, the oceans were anoxic and sulfidic [156]. Importantly, that anoxic and sulfidic condition persisted until ~580 MY ago, the same time when the first animal macrofossils appear in the geological record, with oxygenation of the oceans allowing preexisting and diversified animal lineages to increase in size [157,158].

As such, eukaryotes, which arose some 1.5 Ga ago [159], diversified into their major lineages during anoxic and sulfidic times. It should therefore hardly be surprising (one would think) that diverse eukaryotic lineages have retained oxygen-independent forms of mitochondrial ATP synthesis from their facultatively anaerobic common ancestor [127-129,160-162] or that mitochondrial sulfide utilization is widespread among eukaryotic lineages [163,164]. But the notion that the ability to survive in low oxygen conditions represents a novel and recent evolutionary achievement of eukaryotes, rather than an ancient trait, remains popular with some specialists, and lateral gene transfer from one eukaryote lineage to another is suggested as the mechanism underlying its apparently sparse distribution among modern lineages [165]. What is disturbing is that exactly the same kinds of phyletic patterns (patchy distributions among eukaryote lineages) are usually interpreted not as LGT but, far more reasonably, as evidence for differential loss when it comes to structural proteins [166]. At some point even the staunchest critics of the view that the presence of anaerobic energy metabolism in eukaryotes is ancestral will have to admit that anaerobiosis is not an evolutionary achievement of eukaryotes: it is an ecological specialization. One thinks of *Chlamydomonas *(a green alga) as an oxygen producer with normal O_2_-respiring mitochondria, but after 30 minutes under anaerobic conditions in the dark it starts producing the same end-products of energy metabolism as eukaryotes with hydrogenosomes and using the same genes and enzymes [167,168]. At face value, the theory of descent with modification (as opposed to the theory of lateral transfer) would be a good working hypothesis to explain that observation. *Chlamydomonas *does not have to go out and acquire new genes to survive every time that oxygen levels drop. Just because prokaryotes have lots of LGT involved in their genome evolution, we should not resort to LGT as the default explanation for the origin of shared traits among eukaryotes, because the mechanisms of chromosome heredity differ deeply across the prokaryote-eukaryote divide.

The differences between prokaryotes and eukaryotes in chromosome heredity provide cause for reflection on the origin of the cell cycle: eukaryotes have one, prokaryotes do not. The cell cycle is the basis for cell division and divides the life cycle of a eukaryotic cell into phases where genes are expressed (always in the presence of a nucleus, for reasons relating to spliceosomes [134]), genes are replicated, and genes are distributed among daughters. In eukaryotes with open mitosis, chromosome segregation occurs in the absence of a nuclear membrane [132]. The cell cycle requires the full complement of proteins that make eukaryotes complex [147], hence the cell cycle originated after the origin of mitochondria. Even in Margulis's formulations of endosymbiotic theory, the host for the origin of mitochondria had a nucleus [169]. Related to the cell cycle is the origin of sex, a major transition of early evolution in most people's books [24,25,170], one deserving attention from a fresh perspective.

## Conclusion

There is more to evolution than will fit on any tree. For understanding major transitions in early evolution, we might not need a tree of life at all. But we need to keep our ideas testable with data from genomes or other independent data so as to keep our nose pinned to the grindstone of observations. The very early evolution of life is mostly written in the language of chemistry, some of which is (arguably) still operating today in modern metabolism if we look at the right groups [171-176]. The environments and starting material that the Earth had to offer to fuel early chemistry are variables that only geochemists can reasonably constrain [30,177,178]. One can make a case that acetogens (clostridial firmicutes) and hydrogenotrophic methanogens (euryarchareotes) harbour the ancestral states of microbial physiology in the eubacteria and archaebacteria respectively [179], and some trees are compatible with that view [2,59], as is the distribution of primitive energy-conserving mechanisms [51-54]. But given a transition from the elements on early Earth to replicating cells, the course of prokaryote evolution does not appear to play out along the branches of a phylogenetic tree. For example, Whitman [180] surveyed the biology and diversity of prokaryotes, showing an rRNA tree to discuss matters of classification; but branching orders in that tree play no role in his discussion of diversity or underlying evolutionary processes. If that is the direction we are headed [181], it is not all bad. But having the eukaryotes sitting on one branch in the rRNA tree of life rather than on two, as they should be (or three in the case of plants with their plastids), is far enough off the mark that we should be striving for a better representation of the relationship of eukaryotes to the two kinds of prokaryotes from which they stem.

Eukaryotes are genetic chimaeras and the role of mitochondria in the origin of that chimaerism is apparent [12,103]. Eukaryotes are complex and the pivotal role of mitochondria in the origin of that complexity (as opposed to a pivotal role of phagocytosis) seems increasingly difficult to dispute, for energetic reasons [147]. That leaves little reasonable alternative to the view that the host for the origin of mitochondria was a prokaryote, in the simplest of competing alternatives an archaebacterium [5]. The antiquity of anaerobic energy metabolism and sulfide metabolism among eukaryotes meshes well with newer views of Proterozoic ocean chemistry [155]. A challenge remains in computing networks of genomes that include lateral gene transfers among prokaryotes and the origin of eukaryotes in the same graph. Tracking early evolution without a tree of life affords far more freedom to explore ideas than thinking with a tree in hand. The ideas need to generate predictions and be testable, though, otherwise they are not science. If we check our thoughts too quickly against a tree whose truth nobody can determine anyway, the tree begins to decide which thoughts we may or may not have and which words we may or may not use. Should a tree of life police our thoughts? Working without one is an option.

## Reviewer report 1

Dan Graur, Department of Biology & Biochemistry, University of Houston, Houston, TX 77204-5001, USA

Bill Martin's timely agitprop raises two unrelated methodological issues. The first, which impacts the evolution of prokaryotic genomes and the emergence of the eukaryotic cell, concerns the misuse of binary trees in cases where the phylogenetic relationships cannot be described by bifurcations. This problem is not unique to early evolutionary events. It affects even recent events, such as the evolution of bread wheat, *Triticum aestivum *(an allohexaploid that resulted from two rounds of genome fusion). Such un-binary problems are usually dealt with by the methodology of phylogenetic networks, which should be used whenever reticulate events, such as hybridization, horizontal gene transfer, recombination, fusion, and gene duplication and loss, are believed to be involved. Indeed, the methodology of networks has been used in the past by Bill Martin and his collaborators to great effect. Admittedly, the phylogenetic network methodology is not as yet as mature or as user-friendly as the methodology for producing binary phylogenetic trees, but I regard this obstacle as a minor, easily remediable, one. Of course, phylogenetic networks, like phylogenetic trees, will provide neither proximate nor ultimate causation for the evolutionary occurrences under study. The phylogenetic networks can only serve as topological guidelines of the order and relative importance of the events involved. Beyond the temporal "buchhalterei," scientists will require the type of chemical thinking that Bill Martin advocates in his paper.

### Author's response

*Thanks for the kind words, one of which required a dictionary, and I agree*.

The second issue that Bill Martin raises is infinitely more difficult to solve. It concerns our ability to deal with prebiotic systems in which the rules that govern the process of information transfer from generation to generation are unknown or even unknowable. Of course, as scientists we are not suppose to ever declare unknowability, however, in analogy with NP-complete problems in computer science, we may be mighty close to unknowability by virtue of the immense number of theoretical possibilities and the limited life expectancy of scientists and the Earth they inhabit.

### Author's response

*The referee is certainly correct that the prebiotic evolution topic is at or, in my view, beyond the limit of what is knowable. Indeed, endeavours into the origin of life are unfalsifiable conjecture because even if we constructed a reactor in the laboratory where chemicals go in on the one side and newly arisen life (E. coli with d-amino acids and a different genetic code, for example) pops out the other, we still wouldn't have generated 'proof'of any sort that our microbial ancestors arose that way (the explanandum). We would just have an example that would make a particular narrative more plausible. Aware that a heaping helping of unknowability is hard wired into the problem, we can still pursue the matter with gusto, knowing that the goal is not to find an answer, but instead to structure the problem so that we can talk and write about it in a manner that is meaningful to scientists and to society, which funds our work*.

The major problem with dealing with the origin of life problem is that the boundary conditions are unknown. We need a framework that will allow us to pursue a heuristic program of inquiry. Bill Martin takes a big step in this direction by putting forward a tentative set of conditions that need to be met for life to emerge. Among them are redox conditions, compartmentalization, a certain acceptable range of temperatures, a certain acceptable pH range, and the availability of certain molecules. Furthermore, the prevalence and the locations of sites where such conditions may have existed in the relevant time frame are identified.

### Author's response

*Yes, the boundary conditions are unknown, but if we consider what sorts of conditions life as we know it (the only kind whose origin need concern us) demands and tolerates, then the realm of possible settings for life's origin takes on some very specific constraints. Coupling that with what geologists tell us about conditions on the early Earth constrains the boundary conditions even more*.

Origin-of-Life narratives, however, are difficult to evaluate by traditional molecular evolutionists. For example, the entire edifice of Bill Martin's approach is based on the assumption that "life is about redox chemistry." Will this proclamation turn out to be more useful in the long run than Schrödinger's [182] "life feeds upon negative entropy"?

### Author's response

"Life is about redox chemisty" *leans, of course, on Albert Szent-Györgyi's famous but extremely difficult-to-pinpoint quote*" Life is nothing but an electron looking for a place to rest". *Is this view more useful than Schrödingers's negentropy? It depends on the meaning of 'useful'. Schödinger's 1944 book is credited with having interested in biology folks the calibre of François Jacob, Seymour Benzer, and Maurice Wilkins interested *[183]. *That level of utility, for the book, is difficult to surpass. Regarding the specific concept of negentropy and its utility, the matter is more complicated. Haldane *[184]*surmised: *"The physiologist who can assimilate the idea of that a living organism feeds on negative entropy will come back to the study of metabolism with a slightly novel set of questions to ask. *" It is probably true that most biologists who ever learned introductory thermodynamics noticed right away that life apparently runs contrary to the principle of entropy (increasing disorder). Schrödinger's inferences that the entropy of live matter is lower than that of the foodstuffs from which it was formed appear, however, to have some loopholes. For example, Hansen et al. *[185]*considered in detail the experimental data on the differences of energy (ΔG), enthalpy (ΔH), and entropy (ΔS) between a random arrangement of molecules in solution and live matter of the same composition, with the specific goal of rethinking Schrödinger's negentropy. They conclude that Schrödinger did not sufficiently specify the reaction for which entropy was to be calculated. Specifically they suggest that he conflated the energy required to perform the functions of life (irrelevant to the calculation of entropy) with the energy required for the existence of live matter (the thermodynamically relevant quantity). They argue on the basis of experimental data that the relevant reaction to consider is:*

*"mixture of complex molecules in aqueous solution → live matter of the same composition"*.

*They then present considerable experimental evidence indicating that for that reaction, ΔG, ΔH, and ΔS are all near zero, an important result that demystifies the apparent conflict between life and entropy*.

Forty years ago, I was convinced that Schrödinger's *What is Life? *held the secret to the origin of life. Twenty years later, I concluded that Schrödinger contributed nothing to our understanding of life and its origin. I have since become very reluctant to express either enthusiasm or dislike for any proposal that deals with problems of life's origins.

### Author's response

*I read Schrödinger's book only three years ago. It had almost no influence on me but it is a book that one should have read. Reluctance and wisdom are often related, it is probably wise to lack enthusiasm for questions relating to life's origin*.

Paraphrasing Stephen Sondheim, I must summarize my thoughts thus:

I've gotten through Orgel, Oparin and Miller

Gee, that was fun and a half.

When you've been through Morowitz and Wickramasinghe,

Anything else is a laugh.

### Author's response

*I had the memorable pleasure of meeting Harold Morowitz in 2002 and again in 2006. His insights into the organization of metabolism hold important clues about biochemical evolution and have had a strong influence on my interest in the topic. Perutz complained that Schrödinger ignored chemistry *[183], *but no one can say that about Harold Morowitz*.

## Reviewer report 2

Ford Doolittle, Biochemistry and Molecular Biology, Dalhousie University, 5850 College Street, Halifax, Nova Scotia, Canada B3H 1X5

Not surprisingly, origin and early-evolution-of-life scenarios reflect the disciplinary affiliations of their authors. Nucleic acid chemists are born RNA-worlders, biochemists worry about metabolism, and phylogeneticists think the important question is what genes were in the genome of the last universal common ancestor. Bill Martin, however, is uniquely polymathic in his approach, and thus a challenge for this monomathic reviewer.

### Author's response

*Harold Morowitz is a polymath. My approach is narrow and impatient*.

Mostly I will focus on the phylogenetic and genome evolution aspects of this manuscript. My comments are however discursive. Commendably, Dr. Martin makes it clear at the outset that none of us tree-bashers doubts the basic tree-likeness of the evolution of *multicellular eukaryotes*, although incomplete lineage sorting [186] and hybrid speciation [187]*do *fuzz the twigs. It seems still necessary to emphasize "higher eukaryotic" treeness, and that chimps and bonobos are without any doubt whatever our surviving sister species, so as not to encourage those who believe otherwise (and unfortunately they are globally in ascendance). And I heartily endorse the notion that in abandoning tree-building for the prokaryotic majority of Life and its history, "we do not need to worry immediately about the implications for systematics, which for prokaryotes or phages can hardly be strictly natural anyway".

### Author's response

We certainly agree on that...

But, I'd go further to say we really do not need to worry about prokaryotic systematics at all.

### Author's response

*...but we disagree on that, and we have had this discussion before. No tree of life is one thing, no systematics is quite another. Systematics is defined by some as the branch of biology that deals with classification and nomenclature. If the doctor tells us that its Legionella, that has a meaning with many consequences, whereas if the doctor cannot tell us what it is (because systematics and nomnclature has been banned), we don't have a clue as to whence the cough. I obviously agree that we do not need to worry about **natural **prokaryotic systematics at all; but I maintain that that is fundamentally different from saying that we need no systematics at all*.

Taxa above species are not real things anyway (nor, with exceptions, are species). For multicellular eukaryotes we have long had to acknowledge that the degree of phenotypic or genotypic clustering that defines a species, genus, family or whatever varies with the lineage and the intellectual traditions of those who study it. For prokaryotes there is this arbitariness *and *the further unreality represented by chimerism: there is no unarguably "natural" way to define lineage. And with networks and the other sorts of data representation now emerging to deal with LGT, there is anyway no unarguable *need *to construct any taxa more inclusive than the isolate. All we should want to and can know about any organism is how to predict the states of characters X, Y and Z, given that we know the states of A, B and C. To ask whether it is a member of some species or larger taxonomic group is to ask a question about how we humans organize information, not about biological reality. Systematics was anyway just a short cut for predicting states of X, Y and Z from states of A, B and C, and an aide-memoire. With all biological information digitized, who will need it anymore?

### Author's response

*At higher levels, prokaryote systematics craters like a meteorite. At lower levels, who will need prokaryote systematics? Doctors and patients. Why? Because if the doctor wants to prescribe an antibiotic against Legionella, he has to look in a list that has the query term Legionella, a vocabulary of systematics, in it. To spell Legionella with digital information requires about 43 characters, and Legionella is much easier to remember*.

I am not nearly enough a chemist to critique Dr. Martin's origin scenarios, but too much of a skeptic to accept his "unquestionably yes" as an answer to the question "Can we ever understand anything as complex as the origin of life and early evolution." This is not mere defeatism on my part, but loyalty to a sort of uniformitarianism. I think Dr. Martin would agree with me that the single cellular LUCA to which those who believe in it would want us to trace all genes - or the spatiotemporally dispersed population of gene family cenancestors (each in a different cell) that he and I would replace it with - was a *modern *cell (or population), biochemically and molecular biologically and ecologically. Sure, there were times before that when things were simpler and different, but there is no need to believe that applying biology's time-honored principles of comparison and parsimony will tell us about those times. It is possible to believe that, but we don't need to, and uniformitarianism legislates against it.

### Author's response

*I think the disagreement here has to do with the meaning of "can we ever understand...". If we let understanding mean the power to make experience intelligible by applying concepts and categories, which is my intended meaning, I'll stand by my 'yes'. That assent supposes I take intelligible to mean structuring a problem so that we can talk about it, which is not what the dictionary says. Regarding LUCA, I did not bring the concept of a LUCA into this context or this paper, for the referee's reason: it is like talking about happiness. LUCA means very different things to different people. And how the heck did uniformitarianism (the same processes operating in the past as in the present) creep into this? I think that I am adhering to uniformitarian reasoning in this paper*.

It seems to me safer to suppose that all the contemporary world can provide us in terms of information about origins and early evolution is models. And here the life from the rocks scenario that Dr. Martin has developed along with Mike Russell seem very appealing - though surely underdetermined by the data.

### Author's response

*OK*.

And I have always worried about the microcompartments seen in vents not serving the role that I think compartments need to serve, as differentially replicating targets of natural selection.

### Author's response

*Worries on this aspect are unfounded. The inorganic compartments are territories that can be occupied by organic contents. Selection operates on the contents, but the compartments themselves do not need to physically divide for that selection to occur, obviously. Imagine a honeycomb that spontaneously grows at the edges (the growing system of inorganic microcompartments) and a swarm of parthenogenic bees that lay eggs as fast as they can (replicating contents) and eat slow egglayers. Through natural selection, the fastest bees will prevail*.

As to the greater complexity of eukaryotes and the need for mitochondria to underwrite it, I think it cannot be that simple.

### Author's response

*But what if it is that simple? Of all the traits eukaryotic, which one has the most constructive capability to precipitate the rest? Some have said 'phagocytosis' for 40 years, Nick Lane and I say bioenergetics, and specifically energy per gene. Phagocytosis is a product of complexity, energy is a prerequisite. One can think of it another way: the lack of mitochondria is what has kept prokaryotes simple*.

If I understand what Dr. Martin is saying here, eukaryotes arose in "anoxic and sulfidic times" and we should not be surprised that "diverse eukaryotic lineages have retained oxygen-independent forms of mitochondrial ATP synthesis", on which those taxa formerly known as Archezoa once again must rely.

### Author's response

*Yes, except that many of them have always relied on oxygen-independent ATP synthesis. And while the referee is thinking about microbes, many of the organisms that have retained the capactiy to thrive in anoxic and sulfidic environments are animals (marine invertebrates for example)*.

But then we should be surprised that there are not eukaryotes that have never enjoyed aerobiosis, never had full use of their mitochondria, and thus remained forever simple, because they could not take advantage of this (?) prerequisite to the origin of cell complexity?

### Author's response

*Regarding "full use" (oxygen): As explained in Lane and Martin (2010) mitochondria impart 10,000-100,000 times more energy per gene to their host, whereas oxygen offers a factor of 20. The "full use" of mitochondria (oxygen) is 500 to 5000 times less important in energetic terms (the terms that count) than having mitochondria or not. Put another way, oxygen is only 0.2 to 0.02% as important as mitochondria when it comes to covering the energetic costs of evolutionary inventions*.

Where are these non-complex eukaryotes? All eukaryotes seem to show pretty much the full meal deal. Thus, consistent with my comments on another of the manuscripts in this edition, I think that "fourth domain" scenarios [188] retain their appeal.

### Author's response

*The highly esteemed referee is entitled to enjoy the appeal of fourth domain models. But why stop at four, when we can imagine five or six or more? Fourth domain and similar models (the chronocyte, for example) have something very much in common with radical versions of symbiotic theory: Virtually no evolutionary novelty is attributed to standard evolutionary invention in the history of observable lineages; major novelties are attributed to acquisition only, in fully fledged form, via symbiotic mergers. That leaves all the inventing up to lineages other than the ones that exist, which is problematic. In Margulis's versions, we can at least still observe the suspected donors among modern biota (spirochaetes for example). In contrast, fourth domain scenarios require **all **inventors of eukaryotic traits to go extinct shortly after one single one of their kind acquired the mitochondrion*.

*With all due respect, more distressing with the fourth domain argument from this particular referee is inconsistency in reasoning. The referee says that are no lineages *-- "there is no unarguably "natural" way to define lineage" *above *-- *if we look far enough back in time. Regardless of whether or not that premise is true, it is assumed to be true by the referee. A whole fourth domain, a lineage if there ever was one, and an idealized one at that, is conjured up for the specific purpose of solving a problem (evolving eukaryotic features) that one could less creatively approach with standard principles of evolutionary innovation in the ancestors of observable lineages*.

*How would the referee suggest that the fourth domain finance, in energetic terms, eukaryotic inventions without mitochondria? Lane and Martin (2010) might have put their finger on the sore spot of archezoa and related fourth domain theories: invention and expression of a genome complexity with eukaryotic dimension requires mitochondrial power. The lack of true intermediates in the prokaryote-to-eukaryote transition has a bioenergetic cause*.

*The appearance of the "full meal deal" without leaving intermediate descendants is far better accounted for in the wake of mitochondria, than it is under a fourth domain model, because in the wake of mitochondria, at least the novelties can be afforded energetically. In the fourth domain model they cannot, unless we posit that the fourth domain had some fifth domain mitochondria in order to become complex before one of its many lineages (preprochondriates) lost theirs then acquired the real mitochondria that we see today -- too many assumed lineages, too much imagination*.

*Thus, I disagree that fourth domain models have much appeal, other than to those who would contend that making the leap to the eukaryotic state is asking more than evolutionary theory has to offer. Fingernails are evolutionary inventions, not acquisitions from a lineage of free-living fingernails that became symbionts but whose (n^th ^domain) relatives otherwise became conveniently extinct. Introns, the spliceosome, the endoplasmic reticulum, et cetera, are also most simply assumed to be inventions of eukaryotes, not inheritances from extinct specific-invention-happy donors, until data demand we reason otherwise*.

*If we add a fourth domain, then there is no stopping. We can add more domains and more symbioses at will to solve each eukaryotic invention problem via acquisition, one symbiosis at a time, which allows too much freedom (too little constraint) in inferences about the topic. As regards consistency, we can't deconstruct everyone else's lineages and protect only one (the imaginary fourth) as a host for mitochondria, too*.

## Reviewer report 3

Eugene V. Koonin, NCBI, NLM, NIH, Bethesda, MD 20894, USA

This is a very interesting paper (certainly, fantastic read) that indeed decisively goes beyond the TOL by effectively dismissing the TOL as either an accurate depiction of the evolutionary (outside plants and animals) or a useful tool to study evolution. Without going into much detail, I will state my position on this issue.

### Author's response

*Thanks for the kind words, I will try to be terse, too. My paper deals with more than LGT-ToL, but from the referees's upcooming book *[170]*it seems that we are in broad and general agreement on the coarse outline of things regarding origin of life and origin of eukaryote issues*.

As almost all literature in this field, this article argues against the TOL on the basis of the abundance of "lateral gene transfer" (LGT). And this is I am afraid a contradiction in terms: for any gene transfer to be labelled lateral, there should be a standard of vertical evolution to compare it with. If there is none, then there is no such thing as LGT either, just a tangled web of gene exchanges. But let us think a little deeper about this and ask: what are the nodes in that network of life's evolution? The answer is clear enough: distinct organisms or groups of organisms or lineages (if one takes the view that species do not exist among prokaryotes). And of course we know full well that such lineages exist and they do exchange genes laterally. What does it mean that there distinct lineages of prokaryotes (or any organisms)? Ostensibly, that ensembles of genes (genomes) evolve as coherent, if not immutable, wholes for extended time intervals. In other words, there are substantial fragments of tree-like evolution in the history of the prokaryotic world. So far so good but then it is pointed out that "at depth, the tree of life is not a tree". A statement supported by many observations as any phylogenetic signal certainly deteriorates as one attempts to trace it back to the early days of life's history. Still, is it the case that no signal of coherence between phylogenies of individual genes exists whatsoever? At least one recent study addressed this exact question, and the answer seems to be that the signal persists throughout, however weak it is at the deepest level [189].

### Author's response

*Yes, the deeper we go, the weaker the signals get, for several reasons elaborated there *[189]. *Recalling that folks can just barely agree on the branching orders for mammals (less than 300 MY of evolution) despite abundant homologous molecular data and no LGT that anyone can think of, the existence of any apparent congruence in the Puigbo et al. NUTs (nearly universal trees) is actually the surprise, as Leigh et al. *[190]*show, the methodological issue of ancient phylogenies notwithstanding *[189,191]

So my position is that the TOL is not as dead as it might seem to be and, if interpreted within a flexible conceptual framework, it is not an impediment to evolutionary research at all (as repeatedly suggested in this article). If we reinvent the TOL as a signal of partially congruent evolution of individual genes and try to measure this trend quantitatively, the answer comes out as ~40% tree-like evolution [16]. This is still a minority of the evolutionary processes in the prokaryotic world but a far cry for "a tree of 1%" that the TOL becomes if interpreted simplistically as the tree of concatenated universal (see the critique of [2] in [76] and in this article). I think that the "statistical TOL" reflects two important facets of evolutionary reality:

- A tree is a natural representation of a gene's history [192] as also acknowledged in this article

- Genes do not evolve in isolation but rather in ensembles, and there is coherence in the evolution of such ensembles (genomes) that decays but does not disappear with evolutionary time.

### Author's response

*Yes, genes evolve individually as trees, barring recombination. But I would offer that 40% number needs some hefty qualification. The 40% number comes from an analysis of the NUTs *[191]. *The NUTs, meaning nearly universal trees, are trees for 102 proteins that are present in about 90% of broad sample of prokaryotic genomes *[16,189]. *Recalling that 102 proteins corresponds to about 2-3% of typical prokaryotic genome, the 40% number means that about 40% of about 3% of the genome signal has some vertical component. Forty percent of three percent boils down to a tree of 1.2% which for our present purposes is not at all a far cry from a tree of 1%. We fully agree that towards the tips, prokaryotic evolution is to some extent treelike. But let's keep in mind that E. coli has about 18,000 genes *[193], *of which an individual only has about 4,500. The concept of "vertical" in situations where an individual only has 25% of the genes that its species contains requires more qualifications than we want to discuss here*.

I believe that the "weak TOL" perceived this way is an asset to evolutionary biology rather than an impediment, in part because it is a most convenient - and in my view legitimate - framework for constructing evolutionary scenarios. This position by no account denies the importance of network representation of evolution as in [21]. These are not just useful but necessary perspectives on evolution that are complementary to the tree perspective.

### Author's response

*There is a tree component, and it is at the tips. But my paper is about early evolution, where today's tips have little direct bearing on the issues*.

Finally, I would like to make a few notes on the differences between the tree components of evolution in prokaryotes and in eukaryotes. Surely, it is easier to construct reliable trees for multicellular eukaryotes but is the difference qualitative or quantitative?

### Author's response

*Both, but mostly qualitative*.

I would argue for the latter view.

### Author's response

*OK, let's debate this briefly here*.

Indeed, even in animals and plants, there are substantial parts of the genomes that hardly show much tree-like evolution at any significant phylogenetic depths due to extensive lineage-specific gene loss and even more lineage-specific accumulation of mobile elements. Then, much like in prokaryotes, when one goes deep into the evolutionary past, the tree signal becomes quite faint: indeed, no definitive resolution of the relationships between the eukaryotic supergroups has been reached despite considerable effort [7,194,195]. The pan-eukaryotic core is not 1% like in prokaryotes but is < 10% of the gene repertoires of the large genomes. Yes, evolution of eukaryotes is more tree-like than evolution of prokaryotes (due in large part to meiosis and regular sex in most groups) but the general description is the same: the TOL is not an overarching pattern but a statistical trend and in that form is real and useful.

### Author's response

*Right, and in addition euakryotes boast recurrent genome duplications (ploidization or whole genome duplications) and quick returns to the original haploid or diploid genetics via random loss of homeologues *[196]. *Over time, that leads to massive hidden microparalogy, a phylogenetic resolution problem specific to eukaryotes because prokaryotes lack similar mechanisms. And yes, eukaryote individuals inherit 100%, not 25% of the genes found in the species, and eukaryotes generally do not express their genes during cell division, an issue that relates to the origin of the nucleus, as I think we would argree. For the purposes of this paper (on early evolution), however, eukaryote phylogeny is an issue of the tips, and the tips have a treelike nature, in eukaryotes for sure: on that we agree. Here the debated point in this comment is "how much tree is there deep", and for the most part my essay says, starting with the title "it doesn't really matter", because none of my arguments are based on trees (though some might be congruent with aspects thereof). Given transduction, natural competence, conjugation, gene transfer agents, CRISPRs and the like in prokaryotes vs. "mate with your own kind" inheritance in eukaryotes, I think it is fair to say the following: the difference is quantitative, as you argue, but it is quantitative for qualitative reasons. Thus, can we agree on a remis on this point?*

Yes, definitely.

## Reviewer report 4

Christophe Malaterre

Institut d'Histoire et de Philosophie des Sciences et Techniques

CNRS

France

It appears that the title of William Martin's paper only tells half of the story, namely that part in which it is very convincingly argued that the concept of a genealogical tree is at best inappropriate - and at worst an obstacle - for describing the early evolution of life on Earth (I will call this thesis "argument A"). But there is another half and that other half consists in two distinct yet related arguments about first, a hydrothermal origin of life (argument B), and second, a prokaryote-to-eukaryote transition taking place after the origin of mitochondria (argument C). What really links the three arguments is their relevance to the early evolution of life.

### Author's response

*Yes*.

The paper brings together a wealth of scientific results on each one of the three arguments and on related counter-arguments, and shows how they fit together or do not fit into a larger picture. The article thereby takes the form of a critical review and provides arguments that attempt to increase or lower the credibility of some scientific hypotheses concerning the early evolution of life over others. This is very convincingly done, in particular for the central argument A.

### Author's response

*Thank you*.

On a slightly more critical note though, one might have hoped for a series of converging arguments about what is supposed to be *the *thesis of the paper - namely argument A. Yet, arguments B and C seem to stand somehow on their own, each defending a particular scientific hypothesis, on the origin of life for the first and on the prokaryote-to-eukaryote transition for the second. Of course, the paper could be read as a series of three interconnected theses about early evolution - yet in this case, the background section in particular would need to be adjusted accordingly as the objective of the paper would no longer solely be about the inappropriateness of the tree of life concept for early evolution.

### Author's response

*Everyone has their slant on the ToL. In this paper, and more generally I suppose, my slant is that the role of the ToL in attempting to understand early evolution is peripheral at best. That this is not to everyone's taste I know, and I beg the esteemed referee's indulgence*.

On the other hand, if the objective of the paper is really about establishing argument A - as suggested by the title, summary and background sections - it would be good to show that arguments B and C really contribute to the main argument A, which to a certain extent they do. Yet in this case a difficulty might be that competing scientific hypotheses about the origin of life and the prokaryote-to-eukaryote transition than those argued for in B and C might also be compatible with argument A: for instance, both scientific hypotheses about the prokaryote-to-eukaryote transition that are argued against in argument C involve some form of endosymbiosis that also makes the tree of life concept inappropriate for depicting the genealogical relationships of the first microbial organisms. In other words, instead of arguments B and C in their present form, more general arguments about the existence, during early evolution, of evolutionary processes distinct from the process of natural selection (when this one is interpreted in a narrow sense as "heritable variation in fitness") would fare as well. In particular, one may argue that any variation process that would involve heritable material (genetic or - why not - epigenetic as well) belonging to different types (or species - if such a concept can be precisely defined in the context of early evolution) of organisms would do, from the absorption of small pieces of heritable material (as in the case of lateral gene transfer) to the complete absorption of organisms (as in the case of endosymbiosis).

### Author's response

*A, B and C are mutually consistent but do not necessarily hinge upon one another. I have no more general arguments than these to put forth, other than perhaps the more general argument that chemistry and thermodynamics are important in these issues, which no one in the de-treeing of life community seems to be saying. That leaves me standing relatively alone out in left field, I reckon, which is fine, unless there is lightning on the horizon*.

In any case, in addition to the three arguments that are proposed, the paper is thought provoking in many ways. For me, it also links to the three following questions: (1) What are indeed the evolutionary *processes *at work during the period of early evolution beyond that of natural selection?

### Author's response

*Great question. Regarding "process" in the sense that I think the referee means it (mechanistic cause), I tend to side with Harold Morowitz here that there is a good portion of contingency built in to the chemistry of life, at least as far as the synthesis of building blocks go. Morowitz et al. *[197]*write*

"If one wishes to study biogenesis from the bottom up, the first step is to reason from atoms of the periodic table to those molecules that form the core of biochemistry, those molecules central to the chart of intermediary metabolism in chemoautotrophs. [...] We argue that there is an [...] indication that the chemistry at the core of the metabolic chart is necessary and deterministic and would likely characterize any aqueous carbon-based life anywhere it is found in this universe."

*They wrote that in a paper considering origin of the the kinds of compounds that are common to central metabolism in most organisms. Their conclusion is that chemistry and theromodynamics have the last word. It is thermodynamics that explains why we observe the same molecules in for example the citric acid cycle and its variants across modern cells. They say; citric acid cycle intermediates are not a frozen accident, they are the kinds of compounds one might expect to see in any form of life, for reasons intrinsic to the way carbon, oxygen, and hydrogen tend to form bonds under certain conditions. That is not the same as saying that **all **molecules of life should tend to be spontaneously formed, because much of the chemistry of life is generated by life itself, not by the environment. The amino acids that constitute proteins are a case in point, many of them could well have been the result of biochemical invention *[198], *rather than selected from an organic soup*.

Might it be the case that processes such as drift [199], self-organization [200], tinkering [201] or others indeed prove to be relevant too? (2) What is the *relative significance *of each one of these evolutionary processes? If indeed natural selection is not found to be the major source of evolution during this period, then it implies a strong conceptual change in the way we tend to think about evolution (see for instance the relative significance debate in evolutionary biology [202]). (3) What are the *relationships *between these evolutionary processes and the topology of the genealogical network of the first living organisms? Could it be the case that this topology might be conditioned by some evolutionary processes more than by others? And obviously these questions are not limited to the period of "early evolution" discussed here: they are naturally relevant to *all *of evolution, including what one might call "classic evolution" (see for instance the discussion about Darwinian populations [203]) but also the ealier period of "chemical evolution" that is taken to have preceded that of "early evolution" [204,205].

### Author's response

*Great questions. Drift, self-organization, and tinkering all have prominent places in evolutionary thinking and Dyson, Kauffman, and Jacob *[199-201]*have many insights on these and other topics. But as far as I can tell, they are mostly concerned in those works with principles of the type that Beatty *[202]*also deals with, namely the interplay between natural selection and chance processes en route to complexity at one or the other level. Godfrey-Smith *[203]*operates in much the same realm, with special attention to populations as the unit of selection. Joyce *[205]*and the RNA world provide much evidence for the utility of RNA as an information carrier and a catalyst in the realm of natural selection, while Calvin's book *[204]*for me is the most interesting among the lot. But how to link any of those thoughts with conditions on early Earth and real cells as in the ancestors of the ones we know today? Doolittle did that a couple of times as best the data available at the time would allow *[188,206], *but there was no chemistry or physiology in any of that; that is, there was no consideration of how the first cells were making a living and how that related to chemical processes on early Earth*.

*Dyson [*[199]*, page 53] writes that his mathematical model "...leaves out all the complicated details of real organic chemistry." Mathematics is important, but living things are not made of equations. They are made of molecules and the solvent is water. How the heck does that work and worse, how did it come to be? It requires carbon and energy, that's for sure*.

*Szent Györgyi *[207]*starts his 1957 book with a question "The problem is: how does energy drive life?", which crams quite a big problem into five words, because Szent Györgyi was looking for chemical and molecular mechanisms. In chemical evolution, or the transition from geochemistry to biochemistry, entry to the problem -- for me -- involves reading and reasoning one's way into what energy releasing reactions on the early Earth might be homologous to the energy releasing reactions underpinning carbon and energy metabolism in some groups of modern cells. For what it's worth, I do think that the conections between serpentinization, alkaline hydrothermal vents, accessible electrons in Fe^2+^-containing silicates, CO_2_-reduction, H_2_-production plus CH_4_-production at Lost City, and H_2_-CO_2 _dependent carbon and energy metabolism in some methanogenic euryarchaeotes and some acetogenic clostridia, together with the occurrence of the acetyl-CoA pathway -- and now electron bifurcation *[51-53]*in both groups to conserve energy through cytosolic pools of soluble reduced iron as ferredoxin -- really are telling us something significant about the nature of the most ancient cells, their ecology, and their biology. H_2 _deserves more discussion as a source of energy for early life*.

*All that is to say that the questions posed to me by the referee are too hard to answer: What are the processes? What is their significance? What are their relationships to genealogy? I consider it a compliment to have those questions asked in the context of my essay, but cannot offer satisfactory answers other than some shorthand conjecture: The processes are unspectacular energy-releasing chemical reactions driven by the journey of electrons from serpentinization (Fe^2+^) and H_2 _to CO_2_. Their significance is that we can, in principle, understand those reactions and relate them to energy and carbon metabolism in some modern groups. Their relationships to genealogy among the first cells are that clostridial acetogenesis (eubacteria) and euryarcheal methanogenesis (archaebecteria) would be the ancestral states of microbial physiology whereby the universal common ancestor was not a free-living cell*.

*At any rate, my thanks for the kind and stimulating comments and I beg the referee's endulgence, both for my terse and for my digressive responses*.

Minor comments (not for publication)

### Authors' response

*For detecting so many typing and grammar errors, my sincere gratitude. Thanks very much indeed for such careful reading*.
